# Innovation in animal health under Regulation (EU) 2019/6: Review and recommendations

**DOI:** 10.3389/fvets.2026.1758179

**Published:** 2026-04-13

**Authors:** Klaus Hellmann, Helen Jukes, Matthias Hofer, Jens Peters, Ivo Schmerold, Gabriel Beechinor, Raffaele Bruno, Rory Cooney, Gabriele Braun, Charley Cull, Sven Bergmann, Michael Walkenhorst, Theresa Schlittenlacher, Dejan Cvejic, Juliane Straube, Cornelia Huettinger

**Affiliations:** 1 Klifovet GmbH, Munich, Germany; 2 Stonehaven Cozmix Group, Frauenfeld, Switzerland; 3 BPIvet, Berlin, Germany; 4 European Federation for Pharmaceutical Sciences (EUFEPS), Mannheim, Germany; 5 Health Products Regulatory Authority (HPRA), Dublin, Ireland; 6 Zoetis, Zaventem, Belgium; 7 Veterinary Medicines Directorate (VMD), Addlestone, United Kingdom; 8 Midwest Veterinary Services Inc., Manhattan, KS, United States; 9 City University of Hong Kong, Hong Kong, Hong Kong SAR, China; 10 Research Institute of Organic Agriculture (FiBL), Frick, Switzerland

**Keywords:** animal health, innovation, limited markets, novel therapies, regulation (EU) 2019/6, regulatory science, veterinary development, veterinary medicinal products

## Abstract

This report synthesizes the outcomes of an interdisciplinary conference held in Munich in 2025 to evaluate the impact of the EU Veterinary Medicinal Products Regulation (EU) 2019/6 (1) and associated legislation on innovation in the animal health sector. It highlights the regulatory, scientific, and commercial challenges faced by stakeholders and proposes strategic actions to foster innovation, particularly in the context of limited markets, novel therapies, and sustainability. The report draws on expert presentations and panel discussions, offering a comprehensive overview of the current landscape and a call for regulatory and procedural adaptations to support the development and availability of veterinary medicinal products (VMPs).

## 1 Introduction

In July 2025 an interdisciplinary conference was convened by Argenta Munich to discuss Innovation in Animal Health under Regulation (EU) 2019/6 (1). The invited participants represented the animal health and animal feed industry, national competent regulatory authorities, research institutes, animal health consulting and associated industry groups and federations. The aim of the conference was to share experience from the past 3 years following the implementation of the new legislation on veterinary medicines and medicated feed: Regulation (EU) 2019/6, (EU) 2019/5 and (EU) 2019/4 (1–3) in 2022 and to consider which expectations have been met, especially with respect to innovation, and where there is space for improvement.

The huge efforts, made by the European Commission, the European Medicines Agency (EMA), members of the CVMP (Committee for Veterinary Medicinal Products), numerous working groups and stakeholders, in getting all the delegated and implementing acts and modifications of guidelines implemented are highly appreciated. While a huge volume of work has been managed, it is not surprising that 3 years after implementation, some developments have turned out not to meet all expectations, and that there is a need to consider certain aspects to be adapted, having been overlooked or not anticipated when drafting and implementing these new acts and guidance under high time pressure.

The new European regulations were implemented with the major objective to foster innovation: innovative and protectable technology should find an attractive market to allow return on investment and a consistent and foreseeable regulatory environment.

It should be noted that this report is based on the presentations and actual discussions at the conference in Munich.

## 2 Regulatory framework and innovation—taking different perspectives

### 2.1 Innovation and commercial realities in animal health

The commercial and financial scene of the animal health industry was explained by Matthias Hofer. Data presented demonstrated the strong growth in the animal health sector during the last decade and the industry consolidation which led to an increase in the valuation of the top 10 companies. Two-thirds of the growth has been driven by companion animal products and 70% of this comes from innovative products associated with medications and increasing longevity of pets. However, growth of the pet population and associated spending is levelling out, and on top of the pet area, innovation is now needed to stimulate growth in the livestock sector, which recently has mostly come from new vaccines. Risks posed by zoonotic diseases, the objective to reduce the use of antimicrobials, the rising demand for protein in some regions of the world and the increasing focus on optimization of production and farming practices should act as future drivers, whilst the entry of generic products to the market, decrease in livestock populations and tightening of rules around antimicrobial use are dampeners.

Innovation in companion animal health has traditionally originated from the parent pharmaceutical companies of animal health departments leveraging from developments in human health (e.g., monoclonal antibodies) or crop protection (antiparasitics); however, today small start-ups are frequently innovators in the veterinary market. Potential future growth areas expected are medicines for obesity, oncology and behavioral problems and longevity in pets. In livestock, challenges relate to disease epidemics, antimicrobial and antiparasitic resistance, climate change and a lack of skilled labor affecting husbandry practices. Hence innovation might be seen in vaccines (especially for aquaculture), genetics, nutritional health products, novel parasiticides and methane-reduction products.

However, animal health companies are facing also other challenges: three key commercial challenges were mentioned including the consolidation of veterinary clinics ownership leading to increasing buyer power, changes in go-to-market dynamics such as online retail, and increased competition from generics.

### 2.2 Recap on regulation (EU) 2019/6 and objectives

Gabriel Beechinor reviewed the objectives of Regulation (EU) 2019/6 (1) which was intended to provide incentives to stimulate innovation, but in effect provisions have been limited to extension of data protection periods for VMPs intended for Limited Markets and the specification of requirements for certain types of novel products, e.g., cell therapies. In addition, new IT systems, such as the Union Product Database, will enhance public knowledge of existing products.

Analysis of EMA statistics shows that although a number of new VMPs for companion animals and vaccines for food-producing species have been approved since the Regulation came into effect, there remain some notable gaps, e.g., new antiparasitics for livestock and antimicrobials, probably due to increased controls that impact their use. The current interpretation of the legal texts in respect of VMPs for Limited Markets indicates that the bar has been set relatively high for eligibility to benefit from reduced data requirements under Article 23. EMA data based on their own criteria show that CVMP declined 25 of 57 (44%) requests for data waivers for products classed as Limited Market by mid-2025. Further uncertainty results from the fact that the CVMP’s reflection paper providing guidance on the classification of such products is currently under revision. In addition, the classification of a Limited Market product can change following the original decision, e.g., if new products become available on the market. The classification will be re-evaluated following submission, during the marketing authorization application procedure, before the marketing authorisation is granted. Limited market products do not need to be destined only for an unmet medical need but also have to provide a ‘meaningful advantage’ e.g. in treating a life-threatening disease. This makes this option fitting to very few indications and limits the innovation of niche market products.

Other areas of regulatory increased demands identified by industry include a new ‘mutual cooperation’ procedure for multinational clinical trials, although not compulsory, and requirements for pharmacovigilance including signal management. Information from Access VetMed (4) shows a sharp increase in the amount of time spent by industry particularly on pharmacovigilance duties and on Regulatory Affairs for existing products. Beyond the VMP Regulation, industry must also respond to other challenges such as horizontal legislation (e.g., REACH, package recycling, wastewater), geopolitical issues, climate change and medicines shortages.

The new fee structure implemented for the services of EMA for VMPs has significantly increased registration fees compared to 2024. This increase is likely to serve as a disincentive for the registration of new VMPs for many animal species and indications.

The return of investment is highly dependent on the costs to bring new innovations to market. In the future, new advances might help address this imperative through:

- Consideration of non-animal methods for safety testing
- Adaptation of regulatory requirements
- Development of vaccines for zoonotic and other animal diseases where there is currently an unmet medical need
- Improved accessibility to scientific advice
- Extending options to reduce EMA fees for their services
- Focus on sustainability and animal welfare

In conclusion, innovation is key to advancing veterinary care based on the availability of VMPs on the European market. Regulation (EU) 2019/6 (1) has set the foundation; however, uncertainty and challenges remain. Resilience, adaptability and sustainability are vital to overcome barriers, address disease threats and improve animal health and food security.

### 2.3 Advancing innovation under regulation (EU) 2019/6

Since Regulation (EU) 2019/6 (1) came into effect in 2022, according to Raffaele Bruno, the period for its practical implementation has now been entered and the consequences of the interpretation of the legal text are being observed. There are three key means by which the Regulation can support innovation: providing predictability, remaining aligned and flexible to accommodate innovation, and providing tools to support rewards on investment.

Undoubtedly, the new legislation has had some positive effects on predictability, e.g., in setting out clearer dossier requirements for biological non-immunological VMPs and in explaining the restrictions applicable to persistent, bioaccumulative and toxic substances and antimicrobials. On the contrary, predictability is negatively impacted as certain legal interpretations are only becoming evident 5 years after publication.

One example is how to gain eligibility to reduced data requirements for Limited Market products according to Article 23, where it must be demonstrated that a product meets both of two criteria - that it is intended for a serious disease and meets an unmet medical need. Neither criteria is mentioned in the Regulation, yet they have been implemented later. This requirement was not foreseen during stakeholder review and preliminary discussions, even not during the first evaluations by CVMP.

Another example is in respect of exceptional circumstances authorisations under Article 25: this provision has until now been accepted only for immunologicals. EMA has recently communicated that the Regulation does not preclude this pathway to non-immunological VMPs even if the eligibility criteria are not established yet.

To further encourage innovation, a relevant return on investment has to be assured to the investors. In the highly differentiated animal health markets, with multiple species and minor indications, this argument is even more relevant. While extended data protection periods may be beneficial, they are not as attractive as they are usually exceeded by the patent protection. However, innovation is not limited to introducing new therapies; it can also involve identifying indirect or additional benefits for existing products. Some are explicit objectives of the Regulation and they are also interesting for the industry: allowing these benefits to be included on product labels can offer advantages and enhance returns on investment. Examples include claims for reducing environmental impact or decreasing antimicrobial use. However, progress in this area remains limited. For instance, a recent proposal to include a claim for reduced antimicrobial use was rejected by the EMA because it was considered ‘promotional’, illustrating how the interpretation of existing legal provisions can create barriers to innovation and the objectives of the Regulation.

Drafting regulations that can keep pace with innovation is inherently challenging, given the unpredictable nature of scientific and technological advancements: it is in fact impossible to correctly foresee which new therapies will emerge in the future. Ideally, a regulatory framework should be adaptable, evolving alongside new developments to remain ‘fit for purpose.’ Current regulations identify certain technologies—such as gene therapy, regenerative medicine, tissue engineering, blood product therapy, phage therapy, and products derived from nanotechnology—as ‘novel therapies,’ and provide flexibility in their requirements. While this approach is beneficial, it is important to recognize that innovation is not limited to new technologies; it also encompasses the development of new therapeutic options using established technologies.

The industry is increasingly focused on creating new treatments for chronic conditions, such as chronic kidney disease, oncology, and cardiology. These therapies typically require long-term follow-up to assess efficacy and safety, which can significantly delay market access. One potential solution is to approve therapies based on an initial dataset, with additional information provided post-approval to supplement the evidence base. The conditional license in the USA enables a phased approach to data provision, allowing companies to market drugs that address unmet medical needs while continuing to collect the data required for full approval. It is hypothesized that, although there is no direct equivalent of the conditional licence in the EU Regulation, a similar two-step process may be possible for products classified as novel therapies or those approved under exceptional circumstances: this is because in both cases, the Annex II of Regulation (EU) 2019/6 (1) permits the submission of ‘post-authorisation’ studies following an initial application based on a reduced dataset. Once again, the feasibility of this phased approach under the EU Regulation will depend on the interpretation of current legal provisions made by regulators.

Overall, it is important that the evolving interpretation of legal text does not erode predictability and lead to conflicts with the original objective of the Regulation to support innovation. Support for innovation requires regulatory flexibility and should extend beyond novel technologies and rare/exceptional circumstances also to address new features for existing products and emerging therapeutic areas.

The future of innovation also relies on developing new approaches to generate robust supporting evidence. In this context, real-world data present significant opportunities for both regulators—by enhancing decision-making—and for companies seeking more flexible and relevant data sources. However, integrating real-world data into veterinary regulatory submissions poses several challenges, including issues related to data quality, heterogeneity, and representativeness. Overcoming these obstacles will not be straightforward, but the potential rewards are considerable, as real-world data can offer advantages similar to those seen in human medicine and, in some cases, deliver benefits unique to the veterinary sector (5).

### 2.4 How the UK is supporting innovation

Rory Cooney explained that Veterinary Medicines Regulation (VMR) 2013 [as amended, 2024 (6)], set out the UK controls on veterinary medicines, including their authorization, manufacture, distribution, marketing, supply, prescription and use and that the latest amendments for GB took effect May 2024 to reflect evolving policies, EU exit (Brexit) adjustments, and developments in science and regulation. The UK aims to maintain a modern fit-for-purpose and agile regulatory framework - facilitating development, marketing and availability of VMPs including innovation and novel technologies. The VMR seeks, where the UK government can, to reduce unnecessary divergence between Great Britain and Northern Ireland, to reduce regulatory burden and where appropriate to harmonize technical requirements for data necessary for the authorization of VMPs thus facilitating common development of products to be used across the UK.

Annex 2 to the GB VMRs and respective requirements in most part reflects Annex II to Regulation (EU) 2019/6 (1). Provisions supporting innovation such as the Vaccine Antigen Masterfile, vaccine Platform Technology Master File and Multi-Strain Dossiers are included in GB’s Annex 2 in common with the EU regulation and principles of the relevant EU guidance are accepted. UK Exceptional Marketing Authorizations are streamlined regulatory pathways for urgently needed veterinary medicines (Provisional Marketing Authorizations) or limited market veterinary medicines (Limited Market Authorizations) and are comparable provisions to EU Articles 23 and 25 and principles of the relevant EU guidance are accepted. Provisional marketing authorizations are open to other products in addition to vaccines and an application for a monoclonal antibody for an unmet medical need will shortly be assessed via this route. VMD has already approved bluetongue serotype 3 and highly pathogenic avian influenza vaccines this year as provisional marketing authorization, applying accelerated assessments.

VMD regularly provides pre-submission advice at company meetings free-of-charge and such meetings are recommended to discuss classification and dossier structure for novel therapies. A pilot process is in preparation for a chargeable formal written scientific advice procedure. There is the possibility of collaborating with other global regulators on scientific advice in the future.

The UK VMR are deliberately not overly prescriptive of data requirements for novel therapies in Annex 2 because of the flexibility needed; however, tools are available to facilitate their authorization, e.g., post-authorization studies and risk management plans. Novel products such as monoclonal antibodies, stem cells and DNA vaccines are already authorized on the UK market. Consideration is being given to relevant national guidance for bacteriophage-based products for veterinary use. Relevant guidance from the European Pharmacopoeia, WHO and EU guidance on mRNA vaccine-based products for use in veterinary and human medicines is considered.

The VMD collaborates with regulators internationally, for example through participation at VICH, PIC/S and Codex. The QINTS group is an informal collaboration between the UK, Canada, New Zealand, Australia and the USA. Collaboration with other international regulators and procedures to simultaneously review marketing authorization applications where companies wish to market the same product in multiple jurisdictions are available although interest in vaccines thus far has been limited.

VMD supports industry to carry out field trials in the UK and has approved clinical trials of novel therapy products including virus- and DNA-based actives for cancer therapy and bacteriophage products.

Despite a welcomed increase in applications for marketing authorizations for new vaccines, the UK has experienced vaccine shortages affecting various animal sectors, in common with reports elsewhere globally. VMD is working with stakeholders from government, the pharmaceutical industry, veterinary groups, animal keepers and the wider animal health sector to better understand the problem and identify potential solutions and developing a high-level strategy to address vaccine availability in the UK. VMD aims to set up an innovation hub and support to innovation will be one of the pillars in the approach, which will also draw on methods and expertise of other regulators.

### 2.5 Regulation of novel therapies for human use

Jens Peters gave insight into medicines for human use based on genes, tissues or cells. They are categorized as advanced therapy medicinal products (ATMPs) and fall under an overarching legislative framework, Regulation (EU) 1394/2007 (7). A ‘hospital exemption’ allows national authorization of ATMPs that are not manufactured routinely; otherwise, ATMPs are authorized via mandatory centralized procedure and evaluated by the EMA’s Committee for Advanced Therapies. There are extensive EMA guidelines dedicated to ATMPs, all primarily adopting a risk-based approach.

### 2.6 Clinical trial test permits and use of innovative veterinary medicinal products in clinical trials

Experience with clinical trial test permits was shared by Gabriele Braun. Regulation (EU) 2019/6 exempts VMPs used for research and development. Provisions relating to clinical trials are set out in Article 9 of Regulation (EU) 2019/6 (1), which brings legal enforcement to the requirement for their conduct under VICH Good Clinical Practice [GCP (8)]. Otherwise, application for, and approval of, clinical trials is delegated to national competent authorities (NCAs) in member state(s) where the trial takes place.

Certain other EU legislative frameworks are, however, applied, but differently by different NCAs. Clinical trials are not within the scope of Directive 2010/63/EU (9) on the protection of animals used for scientific purposes. The recital (28) to Regulation (EU) 2019/6 (1) outlines that the principles of 3Rs should nevertheless be applied. Directive 2010/63/EU (9) sets a ‘threshold of severity’ for procedures which is equivalent to ‘the introduction of a needle in accordance with good veterinary practice’. One example is that this criterion appears to be applied by certain member states in respect of procedures carried out within clinical trials; some NCAs even request national animal welfare-based test permits on top of those granted according to Regulation (EU) 2019/6 (1).

Legislation on GMOs is applicable where relevant. In addition, for studies in food-producing species, a preliminary withdrawal period must be proposed, either based on MRLs established under Regulation EU 470/2009 (10), or under the exception provided therein; member states may set provisional withdrawal periods for active ingredients used in clinical studies, where no maximum residue limit is set yet. Some member states additionally require investigational veterinary products to be manufactured according to Good Manufacturing Practice (GMP) and transported to Good Distribution Practices (GDP). At the stage when clinical studies are done, GMP authorization is usually not yet granted. Therefore, such requests can be challenging for new innovative products. Some member states apply stricter rules than currently applicable for human clinical trial supplies.

The approach to clinical trial permits remains unharmonized across member states. The current specific requirements of some NCAs do not support the goal of fostering innovation and pragmatic adaptation is required. The recently offered option to apply for test permits in a “cooperative approach” of NCAs for multinational studies is considered as not meeting the requirement of Regulation (EU) 2019/6 (1): it is highly bureaucratic and takes significantly longer than applying to individual member states.

Logistic challenges arising in such studies apply to both clinical trial supplies and diagnostic samples. Several NCAs require Good Distribution Practices to be applied to clinical trial supplies, although under Regulation (EU) 2019/6 (1), they are exempt. Shipments of diagnostic samples from non-EU countries to EU laboratories require an import license; these are valid for 3 months and must be provided for each shipment. This is highly bureaucratic and restricts the use of one central laboratory for multinational clinical studies, usually recommended according to Annex 2 [Regulation EU 2021/805 (11)]. More positively, since 2025, pharmaceuticals containing GMOs are no longer regarded as dangerous goods under the scope of IATA rules (12).

### 2.7 Overcoming challenges to develop innovative products: import, GMO, animal welfare

VMPs intended for research and development are outside the scope of Regulation (EU) 2019/6 (1) according to Article 2. In this context, Klaus Hellmann stressed that it may not be jointly understood, that pre-clinical trials (under controlled laboratory conditions) and clinical trials (in the field) are defined as part of the R&D process. Clinical trials can be regulated under national legislation and the situation with regard to GMP requirements for investigational veterinary products (IVP) remain unclear; some NCAs request GMP and GDP, no consistency is applied across member states. Information on the requirements at national level has been collated by CMDv and is available on the HMA’s website. GMP Annexes 13 and 16 are relevant to investigational products for human use; this is not applicable to investigational veterinary medicinal products, but some NCAs apply this nevertheless. An import license is considered necessary for IVPs originating from third countries; again, certain NCAs require full EU-GMP requirements for such products imported: GMP and release-testing requirements have been requested by some NCAs, unnecessarily increasing the costs for the conduct of clinical studies in the EU significantly. As a consequence, the timing and resources needed to import IVPs into the EU are significant and should be factored into planning for any such trial. Consistent requirements across borders within the EU would be appreciated.

In conclusion, national requirements for clinical trial supplies in the majority of EU member states are quite burdensome, impacting trial schedules and feasibility. IVPs should be correctly labelled according to Article 9 of the Regulation, but extra national obligations are not helpful. National label requirements should be skipped, English language labels accepted where exclusively vets apply such investigational products, and re-testing of products after import should not be required. It is the responsibility of the sponsors to assure use within clinical studies and representativeness of products tested. Ultimately, all these are considered additional unnecessary burdens, that clearly delay the availability of innovative new and novel veterinary medicines.

GMO-based VMPs, which are typically vaccines or gene therapies, fall under both GMO and the VMP regulation. Directive 2001/18/EC (13) governs ‘deliberate release’ into the environment and requires notification and consent from relevant NCA for GMOs and for VMPs when such GMO is planned to be tested under field conditions of a clinical trial. Although a limited number of test permits have been approved in the EU for studies testing GMO-containing vaccines in food-producing species, mostly under contained conditions or at just one location, all NCAs approached have indicated that they would not grant a test permit for trials testing GMO products in pet animals when they are living at the owner’s home, even if all data on deliberate release are provided. This inhibits the development of these products in the European Union, leaving the regulator with the options of either accepting the dossier without field trials at all or the applicant trying to provide data from other non-EU countries, if at all possible. Public and the regulators should agree on reliability of studies conducted outside of the EU.

As of 2025, the EU is updating the GMO legislation to address new genomic techniques, including methods such as Clustered Regularly Interspaced Short Palindromic Repeats commonly known as CRISPR. This may relate to the exemption from GMO rules for certain new genomic techniques. Current proposals relate to plants only and an extension to VMPs tested in animals would be highly beneficial.

### 2.8 Innovation and requirements for unbiased pre-clinical testing in target animals

With his practical background in running numerous studies as part of the development of VMPs, Charley Cull explained that innovation has led to changes in design and conduct of pre-clinical studies for veterinary medicines, studies which must aid the generation of unbiased data to support the timely development of safe and efficacious products. Facilities undertaking pre-clinical studies need multi-species capability, should be able to handle economically important pathogens and should be accredited to conduct studies according to the required GLP, GCP and animal welfare standards [e.g., as established by the Association for Assessment and Accreditation of Laboratory Animal Care International (AAALAC), (14)]. These requirements necessitate investment in equipment and staff to ensure the necessary technical and scientific expertise is met.

Technologies that utilize AI modelling are emerging. These frequently make use of evolution in biometric monitoring, for example, via ear tags, temperature sensors and force plates to measure pain. *In vivo* models are being developed by the industry in collaboration with clinical research organizations and the regulatory agencies to ensure approval of the protocols and procedures used by the latter. Examples include the use of force plates to measure lameness in cattle and pain associated with castration/tail-docking in pigs or osteoarthritis in dogs. Behavior and anxiety in companion animals can be evaluated using standardized methods, e.g., by recording and recognizing facial expressions and behavior during car rides. The common focus behind many of these models is to allow better use of resources, reduced numbers of animals, and objective and unbiased evaluation following standardized protocols.

### 2.9 New technologies in animal health

Three new biological technologies, developed by Sven Bergmann during his career at a globally well-known research institute in Germany, were presented. All three target a highly important disease in a minor species, such as fish or bees, with an additional benefit of delivering safe produce for human consumption. These are all examples, where the return on investment does not justify the initial costs to obtain a marketing authorization, although these target diseases are of high importance to the specific animal health area. Recent fee increases by EMA are an additional impediment.

One way to control for viral disease in fish has been the use of autogenous vaccines. In many areas of aquaculture, there are no registered VMPs available. The use of autogenous vaccines in the aquaculture industry has been highly successful in controlling endemic disease locally on-farm. One example is the use of an inactivated autogenous vaccine to reduce mortality from red head disease in eel from 95 to 5%; this technology has been used effectively for 21 years to date on eel farms. Considering that nearly all eel farms worldwide have a significant problem with this disease, it is difficult to understand why there is no pathway to authorization of such product for which it is easy to justify the financial investment and, importantly although rescuing more than 50% of the eel population from rearing losses with an obvious benefit for animal welfare.

Another example is the vaccination of koi and carp. A study was conducted in Indonesia to investigate the potential for use of a vaccine against Koi herpes virus in infected parent stock to prevent transmission of the virus to their offspring. Protected eggs from infected parent fish achieved an unprecedented hatching rate of 90% and the offspring remained disease-free when transferred to outdoor ponds on the infected farms for at least 6 months, whilst those from untreated fish succumbed to infection.

The third innovative product that was described was targeting a novel approach to the control of Varroa mites in bees using specific antibodies produced in carp. Options for treatment against Varroa mites in bees are limited – resistance has developed to synthetic chemical acaricides, and organic acids have a low margin of safety. In carp, immunoglobulin M is the main antibody produced and the response develops rapidly. The dorsal shields of Varroa mites were used as the antigen to generate a monospecific and polyclonal antibody response in immunized carp. The antibodies were nebulized into bee hives, where they produce an effect by making the Varroa mites ‘visible’ to the bee immune system. The treatment can be re-administered every 7 days on occasion and has been shown to be highly effective in increasing mite falls significantly when compared to negative controls. The use of animals (carp) in the production of the antibody and requirement for registration as a VMP may be considered drawbacks, but it appears that the benefit–risk is clearly positive considering the residue free honey obtained.

For all three novel technologies, the current regulatory requirements to obtain marketing authorization in Europe are too stringent to justify the financial investment at the time.

### 2.10 Simplified registration of herbal VMPs

An insight into herbal VMPs was given by Michael Walkenhorst and Theresa Schlittenlacher. Of the approximately one million phytochemicals, only about 10–20% are known and very few have been studied. Animals at traditional grazing on diverse pastures are exposed to a broad range of phytochemicals. They are well adapted and have evolved good detoxifying systems. However, although low level dietary intakes are well tolerated, the effects of large amounts of a single phytochemical can be highly uncertain, even toxic. There is extensive evidence that animals use plants for self-medication, and that observation of this behavior by humans led them also to use plants for their own treatment. Use of medicinal plants to treat animals is well documented historically and interest in the use of herbal medicines in livestock and companion animals is increasing again. The fact that herbal medicines are sustainable and biodegradable makes them especially attractive for use in organic farming. Recent clinical trials have also shown potential for herbal VMPs to reduce the use of antibiotics in animals, e.g., for treatment of endometritis in dairy cows.

Although many herbal human medicinal products have been authorized in some EU countries, there are far fewer veterinary herbal medicines. In Germany in 2025, there are more than 800 herbal human medicinal products versus only 17 herbal VMPs. The Regulation (EU) 37/2010 (15) on residues in veterinary medicines includes many plant species with ‘No maximum residue limit required’ status and pasture-grazing animals anyway consume phytochemicals naturally in unknown quantities. Regulation (EU) 2019/6 (1) has introduced the possibility to establish a simplified system for registration of traditional herbal products. Under Article 157, by January 2027, the Commission has to provide a report on the use of these products to treat animals in the EU and is meant to make a legislative proposal for their registration.

A simplified registration system should enable a legal basis to administer herbal VMPs and should be sufficiently pragmatic to encourage companies, including small and medium enterprises, to develop various herbal VMPs with sufficient evidence their being of good quality, safe and effective. Considering the costs involved in generating new supportive data, use should be made of existing knowledge. The true value including the economics associated with the production to GMP, along with the risk to the consumer of exposure to unknown phytochemicals when seen in the context of exposure that occurs naturally, has to be considered and weighed against the opportunity for herbal products that have potential to reduce the frequency of disease and the use of antimicrobials and antiparasitics with its impact on One Health, here antimicrobial resistance.

The MedPlants4Vet network was founded in this context. It is a consortium of over 500 representatives from science, industry and regulatory authorities, established under the EU’s COST Action to gather information on the use of herbal remedies in domestic animals to support the development of simplified registration system.

## 3 Discussion and recommendations for action

The regulatory framework under the preceding Directive 2001/82/EC (16) was already mature with respect to assuring the safety and efficacy of VMPs, although transposition at national level had led to disharmonious implementation in certain areas. The provisions relating to the development of modern IT systems and the ability to future-proof through delegated and implementing acts, which can be more easily updated, are important progressions as a consequence of Regulation (EU) 2019/6 (1). Legal acts are always based on the past and thus the new Regulation could not predict all innovations. Unfortunately, legal interpretations and advice from the Commission have not always been satisfactory for industry or regulators and lead to disharmony within the European Union member states. The inconsistent implementation and thus lack of predictability across member states impact the development and testing of innovative new products, e.g., alternatives to antimicrobials, and the effective use of new regulatory pathways introduced by the new regulation (e.g., limited markets, Vaccine Technology Platforms etc.), major objectives of the regulation. This is a barrier to the development of urgently needed new innovative products in animal health. Although use of real-world evidence is likely to lead to advances in development of human medicines, the amount of data needed to validate endpoints is unlikely to be easily achievable in animals. New approaches in evaluating new technologies and mitigating the benefits and risks associated should also take into account the scenario where no product is available and thus non-tested products are used. The application of post-authorization studies and risk management plans should be used more frequently to support authorizations of innovative VMP and the use of adaptive study design should be explored.

Some GMP-associated assurances requested by NCAs are onerous and do not contribute to clinical trial safety. Although complete EU-GMP is required for a commercial product, test products need to assure representativeness to the commercial product; developing new VMPs takes a long time and the manufacturing to GMP is usually rather a late step within that process, being highly expensive and burdensome. The new Annex 21 of the GMP legislation in Europe, meant to come into force shortly, reflects this well by allowing the use of test products in field studies, that are well characterized, but developed and manufactured according to the state of the development of such innovative product. This should be applied by national competent authorities accordingly leaving the final risk of representativeness with the applicant.

The retrospective nature of the legislation results in industry needing to test the limits of what may be acceptable for innovative products; the associated risk often falls to start-up companies hoping to bring these products to market. The FDA’s approach in the USA allows for more upfront discussion of product development; whereas in the EU, decision is made at the end of the MA procedure based on a thorough evaluation of the complete dossier and finally based on a democratic vote by the CVMP members representing the member states.

In terms of novel methods, there has been initial willingness to embrace new technology, although it seems to have been decided that ‘big data’ is currently not reliable enough in the veterinary sector, with few exemptions. New models are under development, but they must be sufficiently representative to translate to the field situation.

It was noted that there are already simplified systems in place for the registration of VMPs for use in small pets and for homeopathic VMPs, although the latter appears to have had little uptake; for homeopathic VMPs, therapeutic claims are not permitted any more. It remains questionable if the simplified systems are easy enough to justify the investment to authorize such products; it appears that hardly any new such products are registered since the implementation of the new regulation. As indicated in the early presentations and confirmed by the practical examples presented in the last session showcase the huge discrepancy between the public interest to have characterized, for safety and efficacy tested veterinary medicinal products available for all animals and indications and the attractiveness of the current markets especially for limited markets and minor species and indications. In the interest of animals, their welfare, but also for the humans benefiting of the animals, it remains in the public interest to mitigate these interests, as also outlined in the objectives of the Regulation.

As a result of the conference, to support innovation in the EU, and based on the current legislation for veterinary medicinal products, the authors and organizers of the conference, have proposed defined changes which should be considered for implementation in the near future.

Proposals to Competent Authorities for change to current legislation and related guidance.

- Keep to the terms and spirit of Regulation (EU) 2019/6 (1) in its objectives to support innovation, availability of VMPs and reduce antimicrobial resistance. Delegated and Implementing acts should be within the terms of the Regulation, not exceeding its requirements, but opening options available within the Regulation. Where legal interpretation is applied, always consider the balance needed between managing risk and the impact on innovation (predictability, flexibility, costs), being one of the primary objectives of Regulation (EU) 2019/6 (1).
- Use the flexibility of the Regulation to the degree possible to support innovative technologies and limited markets, especially where a need may not have been foreseen at the time of drafting of the legal text. Apply a risk-based approach that acknowledges that hazards cannot be completely avoided. Risk needs to be balanced with benefits but be clear in communication that there is no world/innovation without risk.
- Promote cost-free advice on innovative technologies and regulators could offer a more collaborative approach with industry for innovative products. Open discussion is needed beyond the formal and strict conditions of CVMP’s Scientific Advice procedure, which nevertheless is also valuable in many instances. Consider the EMA fee structure for VMPs, also, in this relationship.
- Take full advantage of the possibility for post-authorization studies and risk-management plans for new therapies. Provide guidance to implement this in the Benefit–Risk evaluation for marketing authorization.
- Open new approaches for innovative technologies including facilitating testing of GMO-based candidate products for veterinary use.
- Apply criteria for eligibility for Limited Market as mentioned in Article 23 and do not extend its limitation further. Fully acknowledge the CVMP decision made on Limited Market status at the time of the CVMP classification for a product and only re-consider the status at the time of authorization if 5 years have elapsed since the classification procedure.
- To benefit animals, humans, and the environment, new products for minor species and indications should be encouraged for the market using a positive, risk-based approach. Such products should be manufactured according to GMP principles, undergo a level of safety testing and demonstrate efficacy (but not in fully controlled studies). This approach would facilitate novel treatments becoming available immediately, contrasting to the often lengthy and uncertain pathway that exists currently based on the strict interpretation of the Regulation.

Proposals to the European Commission and Members of the European Parliament to adapt current legislation at EU level:

- Consider pragmatic approaches to simplify the registration system for herbal VMPs, in particular to address the major hurdles of requirements relating to residues and GMP. When evaluating, consider what can be leveraged from the approach to human herbal medicinal product registration.

Proposals to adapt current procedures under national control:

- Aim towards a simple, quick and aligned set of national requirements for clinical trials, leaving space for flexibility and innovation within the terms of the Regulation, whilst taking a proportionate approach to management of risks in the context of a closely monitored study. An application for a test permit is not intended to be a review for a full dossier but should require a minimum data set under the responsibility of the organization initiating the study, usually the sponsor or its representative.
- Keep to the definition of a VMP and applicability of the Regulation to non-registered products: products for research and development are exempted, thus GMP, GDP and other requirements do not apply. GCP is mandatory as indicated in the Regulation, and the new GMP annexes, where appropriate.
- Avoid adding complexity in national legislation, if required use a harmonized approach within the terms of the regulation and considering the interest of accelerating access to innovation.
- Consider that any innovative product used in clinical studies provides new treatment options to the owners of the animals in that country, with potential specific benefit for all, including humans, the environment, animal owners and, most important, the treated animals themselves. Any registered product is better than routine use of the cascade and the use of products which have not undergone a minimum level of testing to receive a marketing authorization.

Proposals to academia, consultants, industry and investors.

- Where there is an animal or public health need and adequate scientific rationale to support innovation, industry should be prepared to take risks in developing new products, supported by regulators, even if the regulatory framework and guidance are still in their infancy. Regulatory authorities and private organizations provide a wealth of expertise to support innovation and reach the market, including with a return of investment.
- Share your experiences and knowledge and work with well-experienced teams, including regulatory authorities, to accelerate the development of new technologies. Be open to the use of new approaches for testing and evaluating.
- Spend public grants for the development of animal health products diligently, focused on the benefit to the public, animals, humans and the environment. Seek regulatory support to assure targeted research and make responsible use of funds, animals and resources.
- Provide full and timely feedback to consultation on regulatory guidance when offered.
- Make use of existing regulatory support and do not hesitate to give feedback to the current options of the innovation task force, scientific advice and support for small and medium enterprises.
